# Co-Expression and Co-Localization of Cartilage Glycoproteins CHI3L1 and Lubricin in Osteoarthritic Cartilage: Morphological, Immunohistochemical and Gene Expression Profiles

**DOI:** 10.3390/ijms17030359

**Published:** 2016-03-11

**Authors:** Marta Anna Szychlinska, Francesca Maria Trovato, Michelino Di Rosa, Lucia Malaguarnera, Lidia Puzzo, Rosy Leonardi, Paola Castrogiovanni, Giuseppe Musumeci

**Affiliations:** 1 Department of Biomedical and Biotechnological Sciences, Human Anatomy and Histology Section, School of Medicine, University of Catania, Via S. Sofia 87, 95123 Catania, Italy; mszychlinska@unict.it (M.A.S.); pacastro@unict.it (P.C.); 2 Departments of Clinical and Experimental Medicine, Internal Medicine Division, School of Medicine, University of Catania, 95123 Catania, Italy; trovatofrancesca@gmail.com; 3 Department of Biomedical and Biotechnological Sciences, Pathology Section, School of Medicine, University of Catania, 95125 Catania, Italy; mdirosa@unict.it (M.D.R.): lucmal@unict.it (L.M.); 4 Department of Medical and Surgical Sciences and Advanced Technologies, Anatomic Pathology Section, School of Medicine, University of Catania, 95123 Catania, Italy; lipuzzo@unict.it; 5 Department of Medical and Surgical Science, Section of Dentistry, University of Catania, 95123 Catania, Italy; rleonard@unict.it

**Keywords:** *Lubricin*, *CHI3L1*, Osteoarthritis, anterior cruciate ligament transection (ACLT), Immunohistochemistry, mRNA

## Abstract

Osteoarthritis is the most common human arthritis characterized by degeneration of articular cartilage. Several studies reported that levels of human cartilage glycoprotein chitinase 3-like-1 (CHI3L1) are known as a potential marker for the activation of chondrocytes and the progression of Osteoarthritis (OA), whereas lubricin appears to be chondroprotective. The aim of this study was to investigate the co-expression and co-localization of CHI3L1 and lubricin in normal and osteoarthritic rat articular cartilage to correlate their modified expression to a specific grade of OA. Samples of normal and osteoarthritic rat articular cartilage were analyzed by the Kellgren–Lawrence OA severity scores, the Kraus’ modified Mankin score and the Histopathology Osteoarthritis Research Society International (OARSI) system for histomorphometric evaluations, and through *CHI3L1* and *lubricin* gene expression, immunohistochemistry and double immuno-staining analysis. The immunoexpression and the mRNA levels of lubricin increased in normal cartilage and decreased in OA cartilage (normal *vs.* OA, *p* < 0.01). By contrast, the immunoexpression and the mRNA levels of CHI3L1 increased in OA cartilage and decreased in normal cartilage (normal *vs.* OA, *p* < 0.01). Our findings are consistent with reports suggesting that these two glycoproteins are functionally associated with the development of OA and in particular with grade 2/3 of OA, suggesting that in the future they could be helpful to stage the severity and progression of the disease.

## 1. Introduction

Osteoarthritis (OA) is the most common human arthritis characterized by deterioration and loss of articular cartilage [1]. OA is the most prevalent joint condition resulting in physical disability resulting in a high economic burden largely attributable to the effects of disability, co-morbid disease, and the expense of treatment [2]. The main risk factors involved in the pathogenesis of OA are genetics, aging, obesity, injury and biomechanical stress [3]. This condition is associated with progressive hyaline articular cartilage loss, low-grade synovitis and alterations in subchondral bone and periarticular tissues [4].

The causes behind OA development and progression continue to remain largely undefined and understanding the molecular pathogenesis of the disease remains a priority. Recent studies have shown that two glycoproteins may be particularly relevant to OA pathogenesis. The human cartilage glycoprotein chitinase 3-like-1 (CHI3L1) is associated with mediators of inflammation [5,6,7] and cartilage damage involved in the pathogenesis of OA [8]. We have earlier reported an increased expression of this protein in the OA rat model when compared to the control group [9]. Its production has been correlated to joint inflammation and it was suggested that its over-expression could be involved in remodelling and degradation of cartilage in OA joints [9].

Another fundamental aspect of the OA pathophysiological process is represented by the reduced boundary-lubricating ability of synovial fluid [10]. The latter is associated with the reduction of lubricin, one of the major joint lubricants, in both acute and chronic conditions [11]. In this regard lubricin is a glycoprotein that has received considerable attention such as chondroprotective molecule [10,11,12,13]. The association between cartilage, boundary lubrication and evident changes in cartilage tissue after injury has not yet been clearly understood, but considerable evidence from the literature indicates that it may predispose the articular cartilage to degenerate and develop OA [12,14]. Hyper expression of lubricin in transgenic mice has been shown to reduce the severity of both age-related OA as well as in a cruciate ligament transection model of OA [15]. The aim of the present study was to investigate, for the first time, the co-expression and co-localization of CHI3L1 and lubricin in normal and osteoarthritic rat articular cartilage from femoral condyles after anterior cruciate ligament transection (ACLT), morphologically by both immunohistochemistry and double immuno-staining to correlate their modified expression to a specific grade of OA. The purpose of this study was to discover potential roles for both glycoproteins in OA pathophysiology and possibly to improve knowledge in this field in order to find new treatments for inflammatory joint diseases. To strengthen our morphological results we also performed gene expression analyses for both glycoproteins. The experimental model adopted in this study was the induction of a moderate OA in rat by ACLT. This model exhibits a number of characteristics similar to human post-traumatic OA and is widely used in this field of research [9]. In order to evaluate the experimental induction of OA, we performed the radiographic Kellgren–Lawrence OA severity scores, by photographical examination and X-ray microtomography imaging, and the histomorphometric evaluation, by the macroscopic Kraus’ modified Mankin score and the microscopic histopathology Osteoarthritis Research Society International (OARSI) system.

## 2. Results

### 2.1. Radiographic Analysis

**Figure 1:**
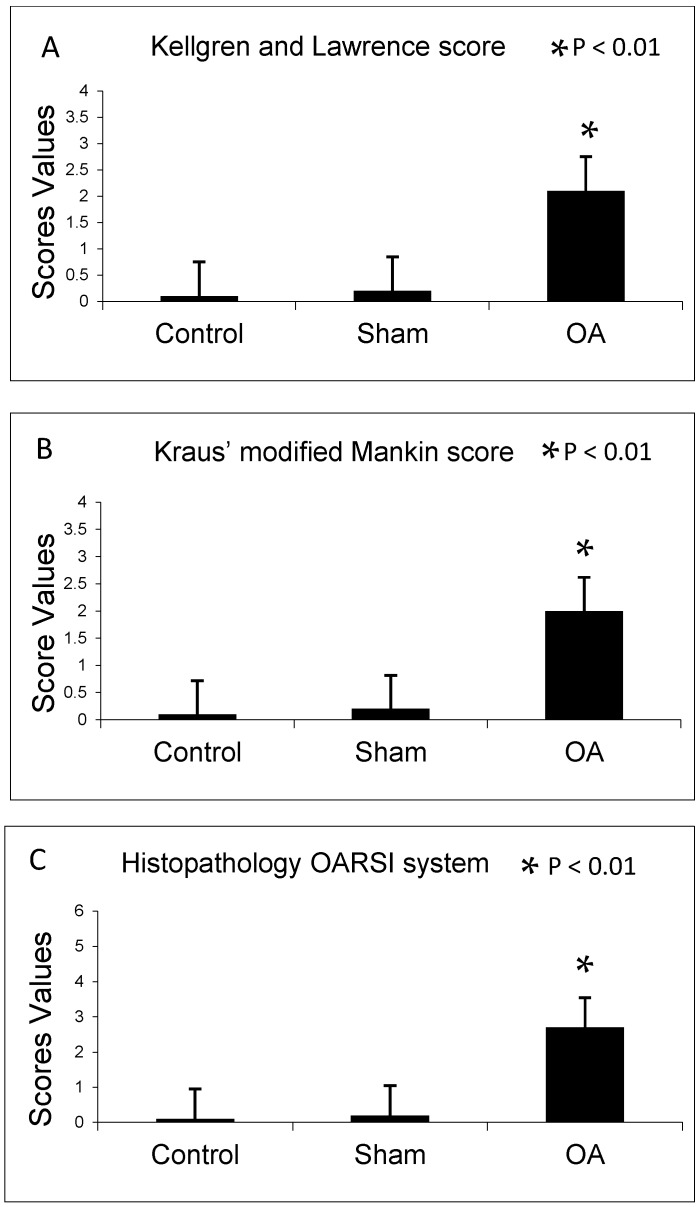
(**A**) Kellgren and Lawrence score among groups; (**B**) Kraus’ modified Mankin score among groups; and (**C**) Histopathology Osteoarthritis Research Society International (OARSI) system among groups. Results are presented as the mean ± SEM. Analysis Of Variance (ANOVA), was used to evaluate the significance of the results. * *p* < 0.01, when compared to the control groups. OA: Osteoarthritis.

In agreement with the Kellgren and Lawrence classification, rats from control and sham groups (without ACLT) showed intact and normal cartilage structure without signs of cartilage degeneration (Kellgren–Lawrence score, Grade 0), while in the OA group (with ACLT) animals showed moderate OA of the knee (Kellgren–Lawrence score, Grade 2) as shown in Figure 1A.

### 2.2. Histomorphometric Analyses

**Figure 2:**
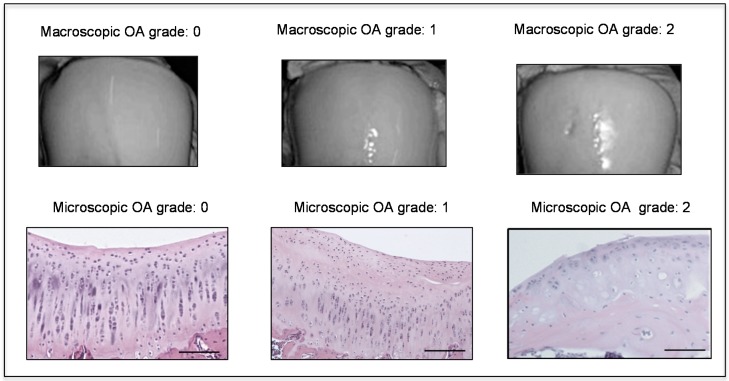
Macroscopic and microscopic articular cartilage degeneration between OA grade 0 to OA grade 2 according to macroscopic Kraus’ modified Mankin score and microscopic histopathology OARSI system, Magnifications ×20, scale bars 100 µm.

The histomorphometric parameters made in both control and sham groups, confirmed the presence of intact and normal cartilage structure without signs of cartilage degeneration indeed both Kraus’ modified Mankin and Histopathology OARSI system scores were 0. In the OA group, instead, cartilage showed more serious pathological changes, consistent with moderate OA with a Kraus’ modified Mankin score of 2 and Histopathology OARSI system score between 2 and 3. Thus, the OA group articular cartilage showed signs of degeneration significantly different from the control groups, as confirmed by Kraus’ modified Mankin score (Figure 1B and Figure 2), and histopathology OARSI system (Figure 1C and Figure 2).

### 2.3. Immunohistochemistry (IHC) Observations

CHI3L1 and lubricin were assessed by immunohistochemical staining in cartilage of all groups. Different patterns of immunopositive cells in the sets of specimens were observed (Table 1).

**Figure 3:**
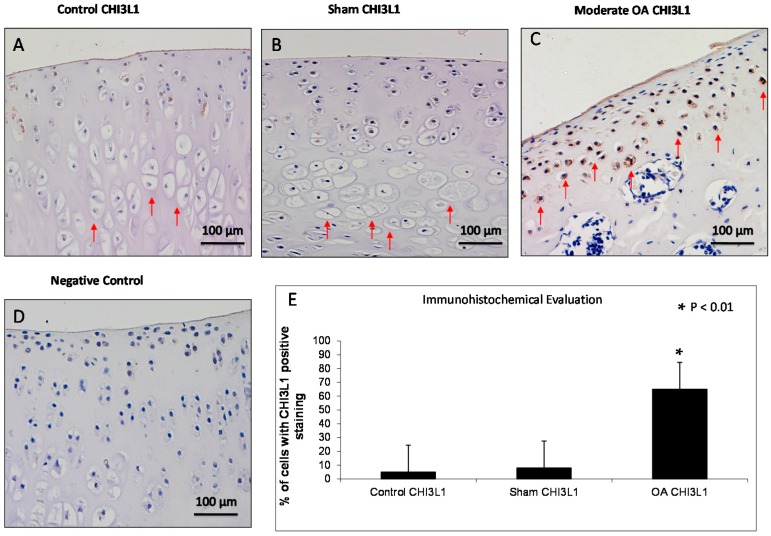
Evaluation of chitinase 3-like-1 (CHI3L1) immunostaining. (**A**,**B**) CHI3L1 immunohistochemistry specimen from control (**A**) and sham (**B**) rat femoral articular cartilage (without anterior cruciate ligament transection (ACLT)) showed a weak/absent (Extent Score (ES) = +; Intensity immunostaining (IS) = 1) immunostaining in cartilage superficial, middle and deep zone in which hypertrophic chondrocytes are evident (red arrows); (**C**) CHI3L1 immunohistochemistry specimen from moderate OA rat femoral articular cartilage (with ACLT) exhibited a strong (ES = +++; IS = 3) immunostaining in middle and deep cartilage zone (red arrows) and a reduction of cartilage thickness of the superficial and the middle zones is evident and in the deep zone the chondrocytes are not hypertrophic and are not arranged in columns; (**D**) The negative control treated with PBS without the primary antibody (CHI3L1) did not show immunostaining (ES = 0; IS = 0). (**A**–**D**) Magnifications ×20; Scale bars: 100 µm; (**E**) Immunohistochemical evaluation graph: percentage of CHI3L1 positive cells out of the total number of cells counted in control groups and in OA group. Results are presented as the mean ± SEM. ANOVA was used to evaluate the significance of the results. * *p* < 0.01, when compared to the control groups.

CHI3L1 overexpression was found in chondrocytes from the OA group mainly in the middle and deep zone of the cartilage rather than the superficial zone, while it was weakly expressed in cartilage from superficial, middle and deep zone of control and sham groups (Figure 3). CHI3L1 immunolabeling was weak/absent (ES = +; IS = 1) in control and sham groups (Figure 3A,B) and was strong (ES = +++; IS = 3) in cartilage from OA group (Figure 3C). The negative control treated with PBS without the primary antibody (CHI3L1) did not show immunostaining (ES = 0; IS = 0) as shown in Figure 3D. The percentage of CHI3L1-positive cells was identified among groups (*p* < 0.01 *vs.* others) as shown in Figure 3E. Interobserver agreement, measured as Cohen’s κ coefficient, was 0.88.

Moderate OA cartilage structural variations included a reduction of cartilage thickness of the superficial and the middle zones, clear deep fissures in the articular surface, and reduction of cells from the superficial, intermediate and deep zone, where chondrocytes are not arranged in columns. The tidemark is not intact in all its extension and the subchondral bone shows fibrillation.

**Figure 4:**
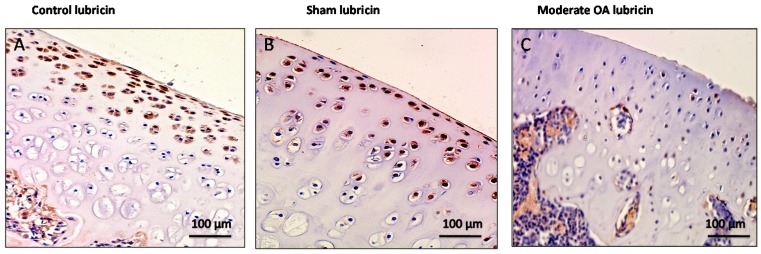

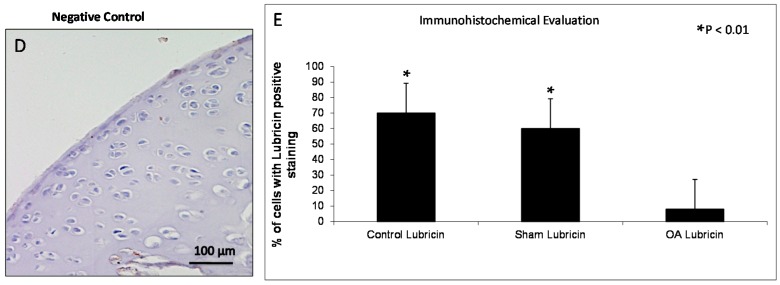
Evaluation of lubricin immunostaining. (**A**,**B**) Lubricin immunohistochemistry specimen from control (**A**) and sham (**B**) cartilage (without ACLT) showed a very strong (ES = +++; IS = 4) immunostaining in chondrocytes from superficial and middle zone of rat femoral articular cartilage; (**C**) Lubricin immunohistochemistry specimen from moderate OA cartilage (with ACLT) showed a weak/absent (ES = +; IS = 1) immunostaining in chondrocytes from rat femoral articular cartilage (superficial, middle and deep zone); (**D**) The negative control treated with PBS without the primary antibody (lubricin) did not show immunostaining (ES = 0; IS = 0). (**A**–**D**) Magnifications ×20; Scale bars: 100 µm; (**E**) Immunohistochemical evaluation graph: percentage of lubricin positive cells out of the total number of cells counted in control groups and in the OA group. Results are presented as the mean ± SEM. ANOVA was used to evaluate the significance of the results. * *p* < 0.01, when compared to the control groups.

In control and in sham groups lubricin overexpression was found mainly in chondrocytes from the superficial and middle zone of the cartilage rather than the deep zone, while it was weakly expressed in cartilage from superficial, middle and deep zone of osteoarthritic cartilage (Figure 4). Lubricin immunolabeling was very strong (ES = +++; IS = 4) in the control and in the sham groups (Figure 4A,B) and was weak/absent (ES = +; IS = 1) in cartilage from the OA group as shown in Figure 4C. The negative control treated with PBS without the primary antibody (lubricin) did not show immunostaining (ES = 0; IS = 0) as shown in Figure 4D. The percentage of lubricin-positive cells was observed among groups (*p* < 0.01 *vs.* others) as shown in Figure 4E. Interobserver agreement, measured as Cohen’s κ coefficient, was 0.92.

### 2.4. Double Immunostaining Observations

**Figure 5:**
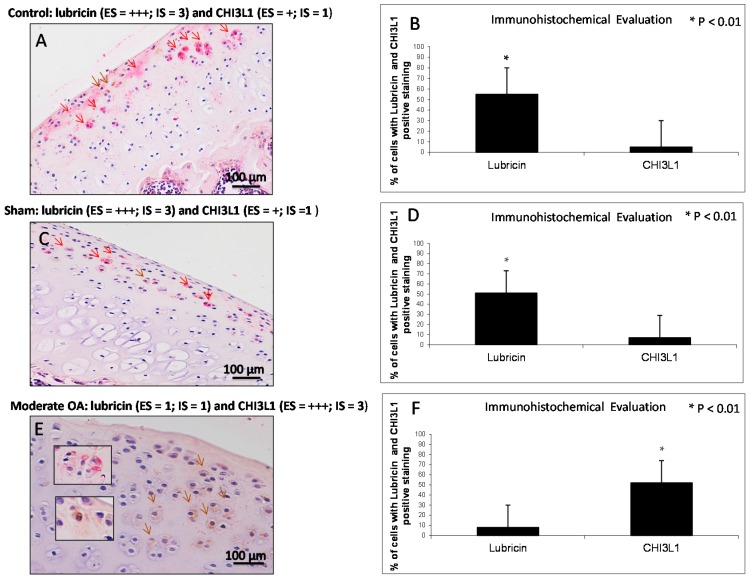
Evaluation of lubricin and CHI3L1 double staining in control and in moderate OA cartilage. (**A**,**C**) In control and in sham groups lubricin immunolabeling was strong (ES = +++; IS = 3, red staining, red arrows), instead the expression of CHI3L1 was weak/absent (ES = +; IS = 1, brown staining, brown arrow); (**E**) In moderate OA, lubricin immunolabeling was weak/absent (ES = +; IS = 1, red staining, red arrows), instead the expression of CHI3L1 was strong (ES = +++; IS = 3, brown staining, brown arrows); (**A**,**C**,**E**) Magnifications ×20; Scale bars: 100 µm; inserts: magnifications ×40; Scale bars: 50 µm; (**B**) Immunohistochemical evaluation graph: percentage of lubricin and CHI3L1-positive cells out of the total number of cells counted in the control group; (**D**) Immunohistochemical evaluation graph: percentage of lubricin and CHI3L1-positive cells out of the total number of cells counted in the sham group; (**F**) Immunohistochemical evaluation graph: percentage of lubricin and CHI3L1-positive cells out of the total number of cells counted in the moderate OA group. Results are presented as the mean ± SEM. Student’s *t* test, was used to evaluate the significance of the results. * *p* < 0.01, when compared lubricin *vs.* CHI3L1.

Double staining was performed with the specific antibodies against lubricin (red) and CHI3L1 (brown) to investigate their expression and to assess their distribution in normal and osteoarthritic articular cartilage tissue. This double stain technique allows us to identify the localization of these two studied proteins. With this technique, we have strengthened and confirmed our previous results, as can be seen from the data presented subsequently. In the control (Figure 5A) and in the sham (Figure 5C) groups, lubricin immunolabeling was strong (ES = +++; IS = 3, red staining), instead the expression of CHI3L1 was weak/absent (ES = +; IS = 1, brown staining). The percentage of lubricin and CHI3L1-positive cells was observed in the control group (lubricin *vs.* CHI3L1, *p* < 0.01) as shown in Figure 5B. Interobserver agreement, measured as Cohen’s κ coefficient, was 0.90. The percentage of lubricin and CHI3L1-positive cells was observed in the sham group (lubricin *vs.* CHI3L1, *p* < 0.01) as shown in Figure 5D. Interobserver agreement, measured as Cohen’s κ coefficient, was 0.86. In the OA group (Figure 5E) lubricin immunolabeling was weak/absent (ES = +; IS = 1, red staining), instead the expression of CHI3L1 was strong (ES = +++; IS = 3, brown staining). The expression of CHI3L1 increases with the intensification of the cartilage damage. The percentage of lubricin and CHI3L1-positive cells was observed in moderate OA (lubricin *vs.* CHI3L1, *p* < 0.01) as shown in Figure 5F. Interobserver agreement, measured as Cohen’s κ coefficient, was 0.96. Lubricin/CHI3L1 ratio was quantified in control and OA groups. The ratio clearly demonstrated the increased expression of lubricin in control cartilage and the increased expression of CHI3L1 in OA cartilage, conversely the decreased expression of lubricin was found in OA cartilage and the decreased expression of CHI3L1 was found in the control cartilage.

### 2.5. Chitinase 3-Like-1 (CHI3L1) and Lubricin mRNA Expression in Osteoarthritic Rat Cartilage Model

**Figure 6:**
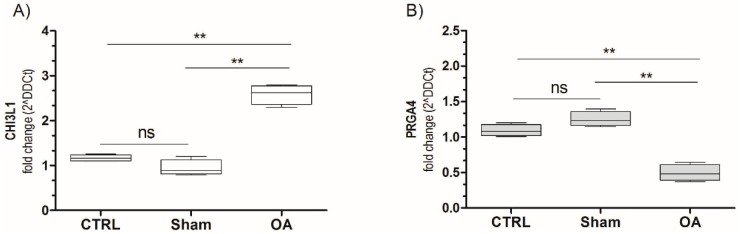
CHI3L1 (**A**) and PRG4 (**B**) mRNA expression in osteoarthritic rat cartilage model. Expression levels of CHI3L1 and PRG4 (lubricin) in cartilage of osteoarthritis rats. Total RNA was extracted as indicated in Materials and Method and CHI3L1/PRG4 expression was measured by real-time PCR. Data are expressed as mean ± SD of at least three independent experiments. ** *p* < 0.001, compared to sham and control. CTRL: control; ns: not significant.

The PCR analysis demonstrated that the CHI3L1 mRNA expression significantly increased in OA cartilage (fold 3.2, *p* < 0.001) compared to the sham and control groups (*p* < 0.001) as shown in Figure 6A. Opposite results were obtained for lubricin, in fact lubricin mRNA expression was significantly reduced in OA cartilage (fold 0.47, *p* < 0.001) compared to the sham and control groups (*p* < 0.001) as shown in Figure 6B. Non-significant results were obtained comparing the sham and the control. These data unequivocally confirm the results obtained by immunohistochemistry, supporting the possible relationship between the opposite CHI3L1- and lubricin-expression and the progression of OA, in particular grade 2/3 of OA.

## 3. Discussion

**Figure 7:**
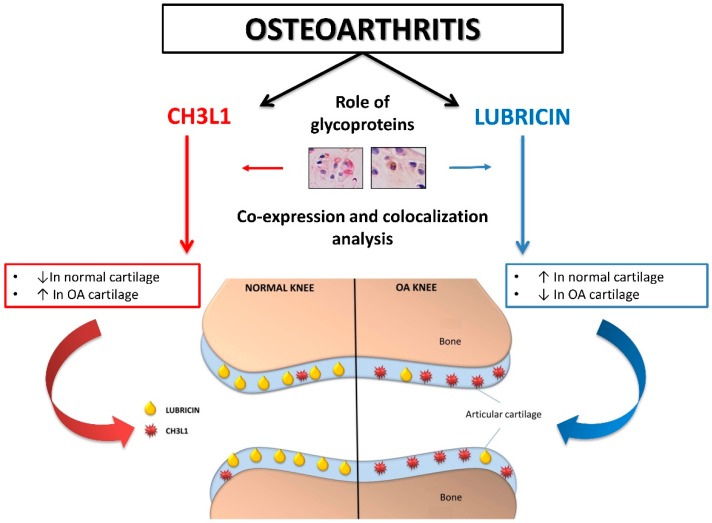
Graphical representation of the involvement of two glycoproteins (CHI3L1 and lubricin) in normal and osteoarthritic articular cartilage. Red arrows (CHI3L1 pathway); Blue arrows (lubricin pathway).

In this study the histomorphometric results in control and sham groups (without ACLT), showed an intact and normal cartilage structure without signs of cartilage degeneration (Kellgren–Lawrence OA severity scores, Grade 0; Kraus’ modified Mankin score, Grade 0; Histopathology OARSI system, Grade 0), whilst in the OA group (with ACLT) cartilage showed more serious pathological changes, as horizontal cleavage tears or flaps and deep lesions, confirming moderate OA (Kellgren–Lawrence OA severity scores, Grade 2; Kraus’ modified Mankin score, Grade 2; Histopathology OARSI system, Grades 2/3). The development of articular degenerative processes in OA group was clear and significantly different from the control groups, as confirmed by the Kellgren–Lawrence OA severity scores, Kraus’ modified Mankin score and histopathology OARSI system. These results were supported by immunohistochemistry, double-staining and mRNA examination. In this study the immunoexpression and the mRNA levels of lubricin increased in normal cartilage and decreased in OA cartilage, while the immunoexpression and the mRNA levels of CHI3L1 increased in OA cartilage and decreased in normal cartilage (Figure 7). We found a negative correlation between the expression of the chondroprotective lubricin and the pro-inflammatory CHI3L1 in normal cartilage and in an experimental model of OA cartilage.

OA is a disease with a high incidence and prevalence, with an expected increase in the number of affected individuals, particularly due to the aging of the population, but also due to the increasing prevalence of obesity and a sedentary lifestyle [16,17]. It is becoming clear that articular tissues other than cartilage play an important role in the process of OA; it is regarded as a whole joint disease. In the last decades the interest of biochemists and biologists focused on glycoproteins and it is well known that there are many biomarkers and glycoproteins involved in the OA pathophysiological process [18,19,20,21,22,23]. This improved attention is partly due to the fact that glycoproteins were revealed to be abundant in living organisms [24]. Many glycoproteins have structural functions, form connective tissues such as collagen, and are used as protective agents and lubricants [24,25,26]. These molecules are composed of a peptide chain with one or more carbohydrate moieties linked *N*-glycosidically or *O*-glycosidically to their constituent protein. According to these structures, glycoproteins are divided in two categories. Fine structural differences within these broader categories, account for the variety of functions among glycoproteins. Specific enzymes regulate glycoprotein synthesis and degradation [24,25,26].

In this study we investigated the co-expression and the co-localization of two fundamental glycoproteins [27,28] involved in cartilage, CHI3L1 and lubricin. CHI3L1, or YKL-40, is a glycoprotein produced by articular chondrocytes, synoviocytes and macrophages. Serum and SF (Synovial fluid) levels of CHI3L1 are increased in inflammatory diseases and correlate with the grade of cartilage degeneration in rheumatoid arthritis. CHI3L1 is a candidate auto antigen in rheumatoid arthritis related to the ability of cells to respond to and cope with variations in their environment [29]. Recently, authors stated that chondrocytes of human osteoarthritic cartilage secrete the inflammation associated chitolectin CHI3L1 [30]. CHI3L1 is a major secretory protein of human chondrocytes in cell culture. CHI3L1 mRNA is not detectable in normal human cartilage, though it is expressed in cartilage from patients with rheumatoid arthritis [31]. Moreover, CHI3L1 levels in SF but not serum were independently and positively related to clinical findings, such as pain and physical disability, in knee OA patients; thus it could represent a potential biomarker of severity of OA [32].

Another important glycoprotein is represented by lubricin. Lubricin is a chondroprotective, mucinous glycoprotein, the product of the *proteoglycan 4* (*PRG4*) gene [33]. It has been found in several tissues including the synovial membranes and SF [34], the superficial zone of articular cartilage [35], tendon and ligament [36], disc and meniscus [37,38]. The lubricin role is maintaining joint integrity. Lubricin is a lubricating glycoprotein present in SF, specifically produced and expressed by articular chondrocytes of the superficial zone. It is recognized as playing a major protective role, preventing cartilage wear and synovial cell adhesion and proliferation and reducing the amount of friction of the articular cartilage surface [39]. It has been also demonstrated that physical activity promotes the expression of lubricin and attenuates the cartilage degeneration process, suggesting again its important role in chondroprotection [40,41,42]. Indeed, the lack of lubricin secretion may be involved in the pathology of OA. Authors demonstrated that considerable negative regulation of lubricin, as well as of other proteoglycans and SF biomarkers, develops in the human knee meniscus and anterior cruciate ligament (ACL) in the acute phase of joint injury, highlighting its involvement in articular injury [38,43]. When recombinant lubricin was injected in injured joints, in a study on animal model of OA, its improved chondroprotection, suggesting its potential use in new approaches for the treatment of OA and other cartilage disease [44,45,46,47,48].

The results of the present research are in accordance with our previous studies [9,35,39]. Here, we demonstrated for the first time the co-expression and the co-localization of these two glycoproteins in *in vivo* normal cartilage, and in an experimental model of OA cartilage. CHI3L1 overexpression was found in chondrocytes from the OA group mainly in the middle and deep zone of the cartilage rather than the superficial zone, while it was weakly expressed in cartilage from the superficial, middle and deep zones of control and sham group cartilage. In contrast, in the control and in the sham groups, lubricin overexpression was found mainly in chondrocytes from the superficial and middle zone of the cartilage rather than the deep zone, while it was weakly expressed in cartilage from superficial, middle and deep zone of osteoarthritic cartilage.

Our results indicate that CHI3L1 and lubricin might be considered as potential natural agents for providing therapeutic protective effects in joint inflammation, and/or may promote cartilage preservation in degenerative disorders of articular cartilage.

The findings of our study suggest that the two glycoproteins, CHI3L1 and lubricin, could be functionally associated with the development of OA, in particular with grade 2/3 of OA evidenced in histomorphometric analysis of our samples, and could be used as biomarker matches, suggesting that in the future they could be helpful to stage the severity and progression of the disease.

## 4. Materials and Methods

### 4.1. Breeding and Housing of Animals

**Figure 8:**
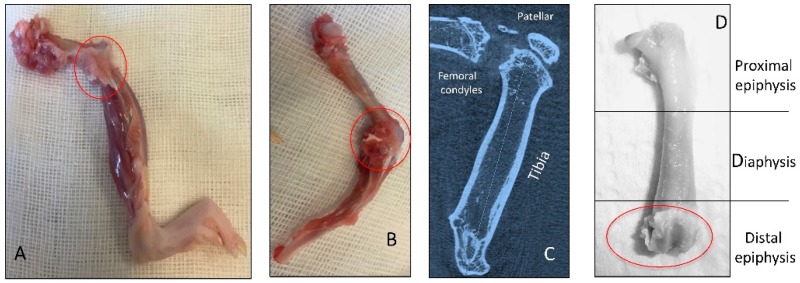
Representation of *in vivo* explanted knee join bones (red circles). (**A**,**B**) Knee OA bones joint after explantation, not cleaned of soft tissues; (**C**) X-ray microtomography imaging of the OA knee bones joint; (**D**) OA femur cleaned of soft tissues.

Thirty 3-month-old healthy male Wistar Outbred Rats (Charles River Laboratories, Milan, Italy), with an average body weight of 160 ± 80 g, were used for this study. Rats were housed in polycarbonate cages (cage dimensions: 10.25″W × 18.75″D × 8″H) at controlled temperature (20–23 °C) and humidity during the whole period of the research, with free access to water and food and photoperiod of 12 h light/dark. Surgical procedures for ACLT were performed in accordance with the method previously described [1,9,49]. The ACLT surgery procedure was made under total anesthesia, 30 mg/kg Zoletil 100 + altadol 5 mg/kg + maintenance mixture of O_2_ and isoflurane 2%–2.5%, (Vibrac, Milan, Italy. The anterior portion of the left hind limb was shaved with an electric clipper, and cleaned with povidone iodine (Sceptre Medical, New Delhi, India). The skin around the knee cap was vertically incised along the medial border of the knee cap. The patella was displaced laterally to expose the anterior cruciate ligament. Then, the anterior cruciate ligament was cut with surgical scissors without injury to the cartilage of the tibia. The patella was then replaced back, and the fascia and skin were closed with a 3–0 polydioxanone suture. A single dose of antibiotic Convenia^®^ 0.1 mL/kg, (Vibrac, Milan, Italy) cream was applied to avoid postoperative infection. After surgery, free cage movement without joint immobilization was permitted to all animals. The 30 animals were divided in two groups: the control group without ACLT (10 rats) and the OA group with ACLT (20 rats). The control group was composed of two subgroups: control normal group (5 rats) without surgical treatment and sham-operated control group (5 rats), receiving the same surgical procedure as the experimental group, without ACLT. The OA group instead consisted of 20 rats submitted to ACLT surgical treatment inducing the OA model. During the experiment the possible suffering of the animals was monitored through the clinical conditions of the animal (fur appearance, weight, consumption of food and water, lameness) evaluated once a day. The animals from all groups at 2 months after the surgical procedures were sacrificed by intracardial Pentothal^®^ injection 30–40 mg/kg (Biochemie, Kundl, Austria); under Furane 2%^®^-narcosis (Abbott Laboratories, Maidenhead, Berks, UK). The pre-operative examinations included physical examination, photographical examination and X-ray microtomography imaging (Figure 8C). The radiographic analyses demonstrating the joint pathology were preliminary to explantation of both femurs that were cleaned from soft tissues and used to perform histomorphometric evaluations (Figure 8A,B,D). Each sample of articular cartilage, from the three groups, was divided in fragments in order to perform histological, immunohistochemical and gene expression analyses. All procedures conformed to the guidelines of the Institutional Animal Care and Use Committee (I.A.C.U.C.) of the University of Catania (Protocol n. 125 of the 1 July 2011, Italian Ministry of Health). The experiments were conducted in accordance with the European Community Council Directive (86/609/EEC) and the Italian Animal Protection Law (116/1992).

### 4.2. Radiographic Analysis

The pre-operative examinations included physical examination, photographical examination and X-ray microtomography imaging (Bruker, Milan, Italy). We chose our samples in accordance with the radiographic OA severity evaluated by the Kellgren and Lawrence score [50] to classify the severity of knee OA that showed radiographic worsening over time. Two blinded investigators (two anatomical morphologists) made the analyses, and the evaluations were assumed correct if no statistically significant difference was showed between the investigators. The Kellgren and Lawrence system provides a score of severity from 0 to 4: Grade 0, no radiographic features of OA are present; Grade 1, doubtful joint space narrowing (JSN) and possible osteophytic lipping; Grade 2, definite osteophytes and possible JSN on anteroposterior weight-bearing radiograph; Grade 3, multiple osteophytes, definite JSN, sclerosis, possible bony deformity; Grade 4, large osteophytes, marked JSN, severe sclerosis and definite bony deformity. The inter-observer variability between two observers for the Kellgren–Lawrence score showed a similar good intra-class correlation coefficient (ICC > 0.84). Repeat scoring by investigators showed very good agreement (ICC > 0.90).

### 4.3. Histomorphometric Analysis

The femurs explantation procedure and the subsequent cleaning of soft tissues was performed as previously described [51]. Samples from all rats (both medial and lateral femoral condyles of untreated and surgically treated animals) were used for the histomorphometric analysis. Histomorphometry was performed with image analysis, Kontron KS 300 software (Kontron Electronics, Eching bei Munchen, Germany) by three blinded investigators (two anatomical morphologists and one histologist). Evaluations were assumed correct if there were no statistically significant differences between the investigators. Fifteen fields randomly selected from each section were analyzed. The semi-quantitative grading criteria of macroscopic Kraus’ modified Mankin score [52,53] and microscopic histopathology OARSI system [54,55] were used. The inter-observer variability among 3 observers for the Mankin system showed a good intra-class correlation coefficient (ICC > 0.92) as for the OARSI system (ICC > 0.89). Repeat scoring by investigators showed very good agreement (ICC > 0.94).

The Kraus’ modified Mankin score provides grades from 0 to 4: Grade 0, normal cartilage; Grade 1, minimal articular damage; Grade 2, articular cartilage damage affecting up to 30% of the articular surface; Grade 3, loss of up to 50% of the articular cartilage; Grade 4, severe loss of cartilage affecting more than 50% of the articular surface.

The Histopathology OARSI system provides grades from 0 to 6: Grade 0, normal articular cartilage; Grade 1, intact surface; Grade 2, surface discontinuity; Grade 3, vertical fissures extending into mid zone; Grade 4, erosion; Grade 5, denudation; Grade 6, deformation.

### 4.4. Histology and Histochemistry Analysis

**Figure 9:**
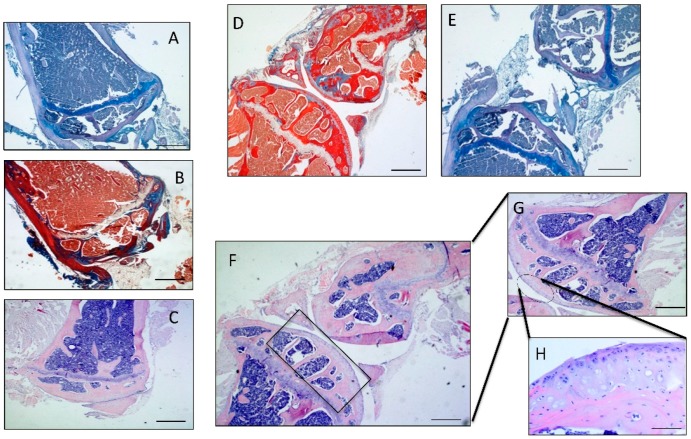
(**A**) Micrograph of the OA distal epiphysis of the femur stained with Toluidine Blue, Magnification ×10; (**B**) Micrograph of the OA distal epiphysis of the femur stained with Masson’s Trichrome, Magnification ×10; (**C**) Micrograph of the OA distal epiphysis of the femur stained with Hematoxylin and Eosin, Magnification ×10; (**D**) Micrograph of the OA knee joint stained with Masson's Trichrome, Magnification ×5; (**E**) Micrograph of the OA knee joint stained with Toluidine Blue, Magnification ×5; (**F**) Micrograph of the OA knee joint stained with Hematoxylin and Eosin, Magnification ×5; (**G**) Magnification of J ×10; (**H**) Magnification ×20, Scale bars: 100 µm.

Some fragments of articular cartilage samples were fixed in 10% neutral buffered-formalin (Bio-Optica, Milan, Italy), following overnight washing and routinely embedded in paraffin as previously described [1]. Samples were positioned in the cassettes in the same direction after wax infiltration. A rotary manual microtome (Leica RM2235, Milan, Italy) was used to cut 4–5 μm thick sections from paraffin blocks that were mounted on silane-coated slides (Menzel-Gläser, Braunschweig, Germany) and stored at room temperature. After dewaxing in xylene, the slides were hydrated using graded ethanol, and stained for histological evaluation by Hematoxylin and Eosin (Figure 9C,F–H) and Masson’s Trichrome (Figure 9B,D) (Bio-Optica) staining for cell identification and the detection of structural alterations. Then the slides were analysed through toluidine blue staining (Figure 9A,E) (Fluka, St. Louis, MO, USA) to assess synthesis of sulfated glycosaminoglycan (GAG) containing proteoglycans (basing on the intensity of staining), in order to evaluate the experimental induction of OA according to the histopathology OARSI system. The samples were examined with a Zeiss Axioplan light microscope (Carl Zeiss, Oberkochen, Germany) and a digital camera (AxioCam MRc5, Carl Zeiss) was used to take the pictures.

### 4.5. Immunohistochemistry (IHC) Analysis

Some fragments of articular cartilage were processed for immunohistochemical analysis was previously described [56]. Briefly, after dewaxing in xylene, the slides were hydrated through graded ethanol and incubated for 30 min in 0.3% H_2_O_2_/methanol to quench endogenous peroxidase activity and then rinsed for 20 min with phosphate-buffered saline (PBS; Bio-Optica). The sections were then heated (5 min × 3) in capped polypropylene slide-holders with citrate buffer (10 mM citric acid, 0.05% Tween 20, pH 6.0; Bio-Optica), using a microwave oven (750 W) to unmask antigenic sites. The blocking step to prevent non-specific binding of the antibody was performed before application of the primary antibody with 5% bovine serum albumin (BSA, Sigma, Milan, Italy) in PBS for 1 h in a moist chamber. After blocking, the sections were incubated overnight at 4 °C with goat polyclonal GP-39 antibody (CHI3L1), work dilution in PBS 1:100 (sc-30465, Santa Cruz Biotechnology, Inc., Dallas, TX, USA) and with rabbit polyclonal anti-lubricin antibody (ab28484; Abcam, Cambridge, UK), diluted 1:100 in PBS (Bio-Optica) for 10 min. Immune complexes were then treated with a biotinylated link antibody (HRP-conjugated anti-goat and anti-rabbit were used as secondary antibodies) and then detected with peroxidase labeled streptavin, both incubated for 10 min at room temperature (LSAB+ System-HRP, K0690, Dako, Denmark). The immunoreaction was visualized by incubating the sections for 2 min in a 0.1% 3,3′-diaminobenzidine and 0.02% hydrogen peroxide solution (DAB substrate Chromogen System; Dako, Denmark). The samples were lightly counterstained with Mayer’s Hematoxylin (Histolab Products AB, Goteborg, Sweden) mounted in GVA mount (Zymed, Laboratories Inc., San Francisco, CA, USA) and observed with an Axioplan Zeiss light microscope (Carl Zeiss) and photographed with a digital camera (AxioCam MRc5, Carl Zeiss).

### 4.6. Double Immuno-Staining Analysis

Double staining was performed with the specific antibodies against lubricin (red) and CHI3L1 (brown) to investigate their expression and to assess their distribution in normal and osteoarthritic articular cartilage tissue. The procedure was performed according to the manufacturer’s instructions using kit EnVision™ G/2 Doublestain System (Dako, Glostrup, Denmark), Rabbit/Mouse DAB+/Permanent Red (K5261; Dako, Glostrup, Denmark). Briefly, the slides were incubated with Dual Endogenous Enzyme Block solution (kit EnVision™ G/2 Doublestain System), containing 0.5% hydrogen peroxide, detergents, enzyme inhibitors and preservative, pH 2, for 5 min before being rinsed with PBS (Bio-Optica) for 5 min. After, the sections were incubated with goat polyclonal GP-39 antibody (CHI3L1; sc-30465, Santa Cruz Biotechnology, Inc.) working dilution 1:100 in PBS. Immune complexes were then treated with dextran polymer conjugated with horseradish peroxidase and affinity-isolated immunoglobulins, Polymer/HRP (kit EnVision™ G/2 Doublestain System), for 10 min. Immunoreactivity was visualized by incubating the sections for 2 min in a 0.1% 3,3′-diaminobenzidine and 0.02% hydrogen peroxide solution (DAB+ Working Solution, prepared by thoroughly mixing 1 mL of DAB+ Substrate Buffer with 1 drop (25–30 μL) of DAB+ Chromogen; kit EnVision™ G/2 Doublestain System). Then the slides were treated with blocking solution, Doublestain Block (kit EnVision™ G/2 Doublestain System), for 3 min, before incubating them with the second rabbit polyclonal anti-lubricin antibody (ab28484; Abcam), diluted 1:100 in PBS (Bio-Optica) for 10 min. The slides were then incubated with dextran polymer coupled with secondary antibodies against goat and rabbit immunoglobulins, Rabbit/Mouse link (kit EnVision™ G/2 Doublestain System), for 10 min and after, with dextran polymer conjugated with alkaline phospatase and affinity-isolated immunoglobulins, Polymer/AP (kit EnVision™ G/2 Doublestain System), for 10 min. At this point, the immunoreactivity for the second antibody was visualized by using the Permanent Red Working Solution, prepared by thoroughly mixing 100 parts of Permanent Red Substrate Buffer with 1 part of Permanent Red Chromogen (kit EnVision™ G/2 Doublestain System), for 10 min. The sections were lightly counterstained with Mayer’s hematoxylin (Histolab Prod-ucts AB, Göteborg, Sweden) and mounted in Dako Glycergel™ Mounting Medium (C0563; Dako, Glostrup, Denmark). The stained slides were observed with an Axioplan Zeiss light microscope (Carl Zeiss) and photographed with a digital camera (AxioCam MRc5, Carl Zeiss).

### 4.7. Evaluation of Immunohistochemistry

The CHI3L1 and lubricin-staining status were identified as either negative or positive. As previously described, immunohistochemical staining was defined positive if brown or red chromogens were detected on the edge of the hematoxylin-stained cell nucleus, within the cytoplasm or in the membrane [56]. Light microscopy was used to evaluate stain intensity and the percentage of immunopositive cells. Intensity of staining (IS) was evaluated on a 4 grades scale (0–4), as following: no detectable staining = 0, weak staining = 1, moderate staining = 2, strong staining = 3, very strong staining = 4. Three investigators (2 anatomical morphologists and one histologist) independently evaluated the percentage of antibodies immunopositive cells through the five categories of Extent Score (ES): <5% (0); 5%–30% (+); 31%–50% (++); 51%–75% (+++), and >75% (++++). Counting was performed under Zeiss Axioplan light microscope at ×200 magnification. If disputes concerning the interpretation occurred, the case was revised to reach a unanimous agreement, as previously described [9]. A digital camera (Canon, Tokyo, Japan) at ×20, ×40 and ×60 magnifications was used to take digital pictures. In this study positive controls, consisted of rat cartilage tissue, and negative control sections, treated with PBS without the primary antibodies, were performed to test the specific reaction of primary antibodies used at a protein level. Positive immunolabeling for antibodies were nuclear/cytoplasmic.

### 4.8. Computerized Morphometric Measurements and Image Analysis

Image analysis software (AxioVision Release 4.8.2-SP2 Software, Carl Zeiss Microscopy GmbH, Jena, Germany), which quantifies the level of staining intensity of positive immunolabelling, was used to calculate the percentage area stained with CHI3L1 and lubricin antibodies in 15 fields, randomly selected from each section. Digital micrographs were taken using the Zeiss Axioplan light microscope (Carl Zeiss, using objective lens of magnification ×20 *i.e.*, total magnification 400) fitted with a digital camera (AxioCam MRc5, Carl Zeiss). Three blinded investigators (2 anatomical morphologists and one histologist) made the evaluations that were assumed to be correct if values have not statistically significant difference [57]. If disputes concerning interpretation occurred, unanimous agreement was reached after sample re-evaluation.

### 4.9. RNA Isolation and Preparation

Some fragments of articular cartilage were immersed in QIAzol (Qiagen, Mississauga, ON, Canada). After homogenization, total RNA was isolated using the Lipid Tissues Mini Kit (Qiagen, Mississauga, ON, Canada), according to the instructions of the manufacturer (Qiagen). RNA quantity was evaluated using the RiboGreen Assay (Molecular Probes, Burlington, ON, Canada), and RNA quality was assessed using an Agilent 2100 Bioanalyzer (Agilent, Palo Alto, CA, USA).

### 4.10. Gene Expression Analysis by Real-Time PCR (qRT-PCR)

Total RNA extracted (500–800 ng per sample) from articular cartilage was reverse-transcribed with RevertAid First Strand cDNA Synthesis kit (Thermo Scientific, Milan, Italy) in a 20 μL reaction solution. Quantitative RT-PCR was performed using one-twentieth of the RT products and platinum SYBR Green qPCR SuperMix UDG with Rox (Invitrogen Life Technologies, Milan, Italy). The primers used are shown in Table 2. The reaction was followed by a melting curve protocol according to the specifications of the ABI 7900 instrument (Applied Biosystems, Foster City, CA, USA). Rat β-Actin (ACTB) was used as a housekeeping gene for normalization. Data are presented as mean ± SD of at least three independent experiments. Differences were analyzed by Student’s *t* test, with *p* < 0.01 being considered statistically significant.

### 4.11. Statistical Analysis

Statistical analysis was performed using GraphPad Instat^®^ Biostatistics version 3.0 software (GraphPad Software, Inc., La Jolla, CA, USA). Data were tested for normality with the Kolmogorov–Smirnov test. All variables were normally distributed. Student’s *t* test was used for comparisons between two means, while analysis of variance (ANOVA) and Bonferroni’s test were used for comparison between more than two groups. *p*-values of less than 0.05 (*p* < 0.05) was considered statistically significant; *p*-values of less than 0.01 (*p* < 0.01) was considered highly statistically significant; *p*-values of less than 0.001 (*p* < 0.001) was considered extremely statistically significant. Data are presented as the mean ± SEM. Cohen’s κ was applied to measure the agreement between the two-blinded observers and averaged to evaluate overall agreement.

## 5. Conclusions

The therapeutic management of OA remains a challenge for physicians. In this setting the basic sciences are involved in finding information regarding the pathophysiological process of this common and severe disease. In this study we highlighted a possible cross-link between two pathological aspects of the inflammatory process and the altered lubricating ability occurring in the joint tissue following chronic disease, such as OA. Our findings might motivate further studies about the link between the two important aspects of OA, inflammatory process through the study of CHI3L1 and lubricating ability through the study of lubricin. However, further studies are needed to understand the exact mechanisms in which these molecules are involved in order to design therapies or treatments to prevent or attenuate the osteoarthritic process.

## Figures and Tables

**Table 1:** Evaluation of lubricin and chitinase 3-like-1 (CHI3L1) immunostaining. Intensity immunostaining (IS); Percentage of immunopositive cells expressed by Extent Score (ES): <5% (0); 5%–30% (+); 31%–50% (++); 51%–75% (+++), and >75% (++++). ACLT: anterior cruciate ligament transection; OA: Osteoarthritis.

Groups	Lubricin	CHI3L1
Control rats without ACLT	Very strong immunostaining (ES = +++; IS = 4)	Weak/absent immunostaining (ES = +; IS = 1)
Sham operated control rats	Very strong immunostaining (ES = +++; IS = 4)	Weak/absent immunostaining (ES = +; IS = 1)
Experimental rats with ACLT (OA)	Weak/absent immunostaining (ES = +; IS = 1)	Strong immunostaining (ES = +++; IS = 3)

**Table 2:** Primers used in gene expression analysis by real-time PCR (qRT-PCR). *PRG4*: proteoglycan 4; ACTB: β-Actin; Ta: thymine and adenine.

Primers	Forward	Reverse	Ta	Size
*PRG4*	CTACAACAGCTTCTGCGAAGAA	GATTTGGGTGAACGTTTGGTGG	60	117
*CHI3L1*	GAGCTGCTTCCCAGATGCCC	CATGCCATACAGGGTTACGTC	60	121
*ACTB*	CATGTACGTAGCCATCCAGG	CTCTCAGCTGTGGTGGTGAA	57	225

## Abbreviations

OA	Osteoarthritis
GP-39	Glycoprotein 39
CHI3L1 YKL-40	Chitinase 3-like-1
SF	Synovial fluid
SZP	Superficial zone protein
PRG4	Proteoglycan 4
ACLT	Anterior cruciate ligament transection
